# Harlequin Syndrome in a Case of Toxic Goitre: A Rare Association

**DOI:** 10.1155/2011/293076

**Published:** 2011-07-12

**Authors:** P. V. Pradeep, Ajith K. Benede, Skandha S. Harshita, B. Jayashree

**Affiliations:** ^1^Department of Endocrine Surgery, Narayana Medical College & Superspeciality Hospital, Chinthareddypalem, Nellore, Andhra Pradesh 524002, India; ^2^Department of Anesthesiology, Narayana Medical College & Superspeciality Hospital, Chinthareddypalem, Nellore, Andhra Pradesh 524002, India

## Abstract

Harlequin syndrome (HS) is known to be associated with conditions like brain stem infarcts and superior mediastinal neurinoma. However, it has not been reported in association with autoimmune hyperthyroidism. We report a case of exacerbation of unilateral sweating in a patient with HS following the onset of toxic goitre. Previous reports have suggested that a tortuous inferior thyroid artery can produce neurovascular compression of the sympathetic chain which was not observed in our patient. Autoimmune aetiology for HS needs to be explored. Increased sweating in hyperthyroid patients needs to be assessed properly so as to prognosticate appropriately.

## 1. Introduction

Harlequin syndrome (HS) is characterized by unilateral facial flushing and sweating induced by exercise or heat [1]. It is considered a benign, idiopathic condition causing failure of the upper thoracic sympathetic chain with sparing of the first (oculomotor) thoracic segment, wherein hard physical working situations are the precipitants [2]. It is known to be associated with brain stem infarcts, superior mediastinal neurinoma, internal jugular vein catheterization, and carotid artery dissection. Increased sweating is a well-described phenomenon in the hyperthyroid patients which resolves after the definitive treatment. We report a case of Harlequin syndrome associated with hyperthyroidism (toxic multinodular goitre). In these cases, it is important that the physician rules out etiological factors like mediastinal tumour, and the patient needs to be advised about the syndrome persisting despite surgery. We present a case of exacerbated Harlequin syndrome in a case of hyperthyroidism which has not been reported in English literature.

## 2. Case History

**Figure 1:**
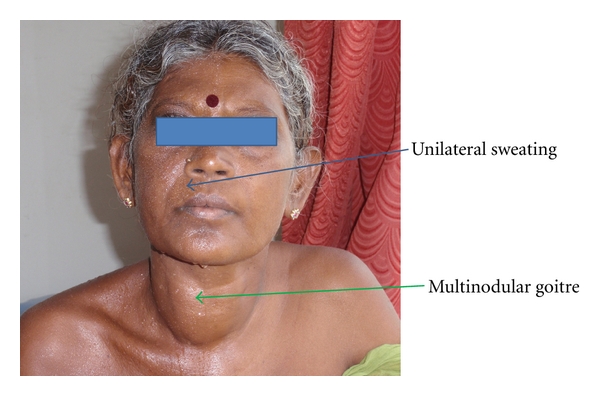
It reveals the unilateral nature of sweating and the multinodular goitre.

A 36-year-old lady presented to us with anterior neck swelling and symptoms of hyperthyroidism since five years. On examination, she had tachycardia, tremors of the extremities, increased sweating, and a large multinodular goitre. Her FT3, FT4, and TSH levels were 5.9 pg/mL (2.5–3.9), 3.1 ng/dL (0.8–2), and 0.01 (0.3–5 mIU/L), respectively. Ultrasound of the thyroid revealed multiple nodules in both lobes of the thyroid. The right lobe was 6.8 × 3.2 × 2.2 cm and the left lobe was 5.7 × 2.8 × 2 cm. Serum thyroperoxidase antibody level was 10.4 IU/mL (normal values: <9 IU/mL), and TSH receptor antibodies were 8.1 U/L (>1.5 U/L positive). Euthyroid state was attained with 120 mg of Carbimazole and 60 mg of Propranolol. Since she had toxic goitre and needed large doses of antithyroid medication, it was decided to proceed with a total thyroidectomy. At the initial evaluation, the patient complained of generalized sweating, but once the toxicity was controlled with antithyroid medications, the lack of sweating on the left half of the face was revealed (Figure 1). 

**Figure 2:**
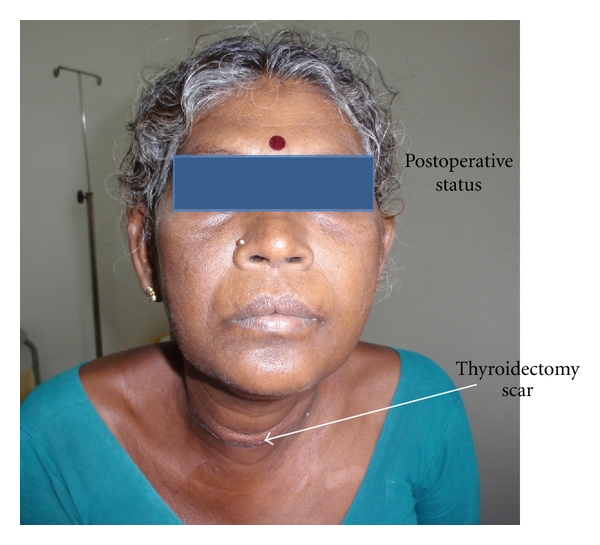
It depicts the postoperative status with the thyroidectomy scar.

The patient had also felt that she is sweating more on the right side of the face which appeared six months after the thyroid swelling. There was no associated Horner's syndrome. MRI of the neck and superior mediastinum revealed no lesions. At surgery, the right lobe of thyroid measured 6.5 × 3 × 3.5 cm, left lobe 6 × 3 × 3.2 cm and contained multiple nodules. The inferior thyroid artery was visualized on both sides. The left inferior thyroid artery was longer and tortuous in course when compared to the right side. After the surgery at 1.5 years of followup, Harlequin syndrome persisted (Figure 2). She had no signs of hypocalcemia or voice changes. 

## 3. Discussion

After the initial description of Harlequin syndrome in 1998 by Lance et al., numerous such case reports and reviews have been published [1]. Harlequin syndrome is considered a benign, idiopathic condition caused by failure of the upper thoracic sympathetic chain with sparing of the first (oculomotor) thoracic segment, wherein hard physical working situations are the precipitants [1, 2]. It is also associated with other autonomic system disturbances, namely, acquired and congenital Horner's syndrome, stroke, and mediastinal tumours [1]. Torsional occlusion of the anterior radicular artery was proposed as the root cause by Lance et al. [1].

**Figure 3:**
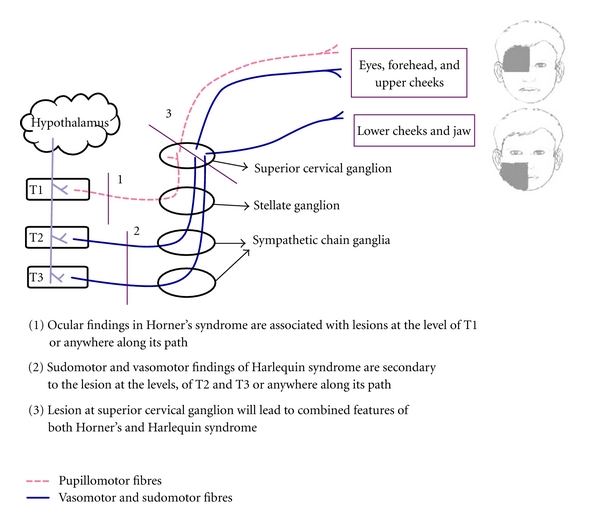
It depicts sympathetic innervation of the face and clinical manifestations due to nerve injury.

The sympathetic outflow pathway originates from the hypothalamus (first neurons) and synapse in the lateral horn of spinal cord (second neurons). Sudomotor and vasomotor fibers innervating the face leave the spinal cord with the ventral roots T2, T3 and travel along the sympathetic chain to the superior cervical ganglion and synapse with the third neurons (Figure 3). Oculosympathetic neurons originate at T1 and travel along the same path. On the basis of the pattern of involvement of the face and presence or absence of Horner's syndrome, localization of the site of the lesion can be performed [2]. If the entire forehead, cheek, and chin are affected, it indicates that the lesion is below the bifurcation of the common carotid artery (CCA). The fibers supplying the medial forehead and nose travel along the internal carotid artery, and if these areas are involved, it indicates that the lesion is distal to the division of the CCA. Similarly, in patients with a central lesion, emotional sweating will not be seen [2]. Intact sympathetic innervations to the ipsilateral upper arm indicate that the lesion is located distally to the stellate ganglion. 

Excessive sweating is very common in patients with hyperthyroidism. This usually disappears after the definitive procedure like radio-iodine therapy or total thyroidectomy. To start with, our patient had generalized increase in sweating due to the hyperthyroidism; however, after the toxic features were controlled, the unilateral nature of the sweating became obvious. In a patient with unilateral sweating, the physician usually suspects Horner's syndrome if the opposite side has ptosis, miosis, and enophthalmos. In cases like the present one where there is absence of enopthalmos and miosis, the physician is most likely to label this sweating as part of hyperthyroidism. In such cases, the unilateral sweating will persist postoperatively, and hence the patient has to be made aware of this fact prior to surgery. Harlequin syndrome may or may not be associated with Horner's syndrome. This is due to the fact that ocular findings in Horner's syndrome are associated with lesions at the level of T1, whereas the sudomotor and vasomotor findings of Harlequin syndrome are associated with the lesion at the levels of T2 and T3 [3, 4].

**Figure 4:**
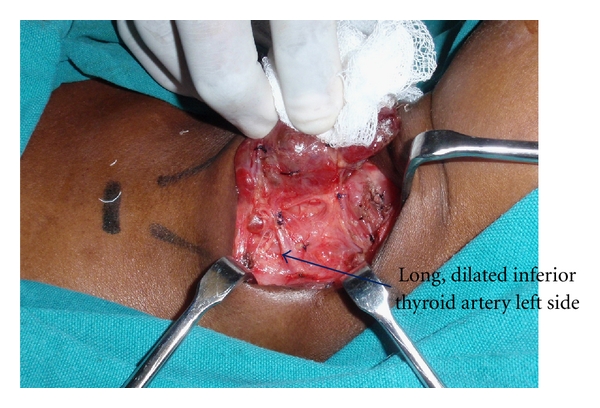
It depicts the long dilated inferior thyroid artery of the left side.

It has been suggested that since the inferior thyroid artery crosses the sympathetic chain in the neck between the stellate and superior cervical ganglion, a tortuous inferior thyroid artery can produce neurovascular compression of the sympathetic chain [5]. Wasner et al. [5] had suggested tortuous inferior thyroid artery as a possible mechanism for Harlequin syndrome in one of their euthyroid patients. In patients with hyperthyroidism, the inferior and superior thyroid arteries enlarge in calibre and become tortuous. Since our patient developed the symptoms of Harlequin six months after developing hyperthyroidism, we explored the possibility of this theory as the cause of Harlequin. Even though during surgery, it was noticed that the inferior thyroid artery of the left side was larger in diameter and had a tortuous course (Figure 4), the Harlequin syndrome has persisted after the total thyroidectomy (1-year postoperative followup). During the thyroidectomy, the main trunk of the inferior thyroid artery was ligated and divided. After thyroidectomy, in cases of hyperthyroidism, the remaining part of the inferior thyroid artery will decrease in calibre and size and therefore may not compress the sympathetic trunk any more. Hence, we feel that dilated tortuous inferior thyroid artery as a possible mechanism may not hold true. However, it is also possible that the HS has persisted even after surgery in our case because of the severe irreversible axonal injury secondary to long-standing compression by the inferior thyroid artery (5 years). 

Since toxic goitres have an autoimmune aetiology, a possible autoimmune pathophysiology should also be considered, and more research is needed in this direction. In our patient, the thyroperoxidase and TSH receptor antibodies were positive suggesting autoimmune origin of the goitre. However, the limitation of this hypothesis is the fact that the right side sympathetic chain was spared. The fact that HS appeared six months after the onset of hyperthyroidism may also be against autoimmune theory. 

Perioperative occurrence of Harlequin lasting for five hours postoperatively has been described after high-volume paravertebral block at T3/T4 level [4] and also after difficult neck mass excision [6]. A case of HS was described in a patient with mediastinal neurinoma which persisted in spite of the resection [7]. Burlacu and Buggy [8] explained that the normal or excessive vasodilatory, thermoregulatory response to heat or emotion on the erythematous (right side as in our case) and relative pallor on the left side was most likely due to differential sympathetic blockade [8]. Contralateral sympathectomy has been suggested as treatment for patients who experience severe social embarrassment as a result of the sweating [5]. 

To conclude, Harlequin syndrome is a rare but worrying symptom for the patient and physicians. Most cases are benign in nature, however, without any specific treatment. Harlequin sign should alert the physician about the coexistence of Horner's syndrome, and appropriate investigations should be done to rule out sinister causes like mediastinal masses, carotid artery dissection, and so forth. Increased sweating in hyperthyroid patients needs to be assessed properly so as to prognosticate appropriately.
